# 2,2′-[2,4-Bis(naphthalen-1-yl)cyclo­butane-1,3-di­yl]bis­(1-methyl­pyridinium) bis­(4-chloro­benzene­sulfonate): thermal-induced [2 + 2] cyclo­addition reaction of a heterostilbene

**DOI:** 10.1107/S160053681400645X

**Published:** 2014-04-02

**Authors:** Suchada Chantrapromma, Kullapa Chanawanno, Nawong Boonnak, Hoong-Kun Fun

**Affiliations:** a Department of Chemistry, Faculty of Science, Prince of Songkla University, Hat-Yai, Songkhla 90112, Thailand; b Faculty of Traditional Thai Medicine, Prince of Songkla University, Hat-Yai, Songkhla 90112, Thailand; c X-ray Crystallography Unit, School of Physics, Universiti Sains Malaysia, 11800 USM, Penang, Malaysia

## Abstract

The asymmetric unit of the title salt, C_36_H_32_N_2_^2+^·2C_6_H_4_ClO_3_S^−^, consists of one anion and one half-cation, the other half being generated by inversion symmetry. The dihedral angle between the pyridinium ring and the napthalene ring system in the asymmetric unit is 42.86 (6)°. In the crystal, cations and anions are linked by weak C—H⋯O inter­actions into chains along [010]. Adjacent chains are further arranged in an anti­parallel manner into sheets parallel to the *bc* plane. π–π inter­actions are observed involving the cations, with centroid–centroid distances of 3.7664 (8) and 3.8553 (8) Å.

## Related literature   

For background to stibene and [2 + 2] photodimerization, see: Chanawanno *et al.* (2010 ▶); Chantrapromma *et al.* (2007 ▶); Papaefsta­thiou *et al.* (2002 ▶); Ruanwas *et al.* (2010 ▶); Yayli *et al.* (2004 ▶); Zhang *et al.* (2013 ▶). For related structures, see: Chantrapromma *et al.* (2012 ▶); Fun, Chanawanno & Chantrapromma (2009 ▶); Fun, Surasit *et al.* (2009 ▶). For bond-length data, see: Allen *et al.* (1987 ▶). For the stability of the temperature controller used in the data collection, see: Cosier & Glazer (1986 ▶).
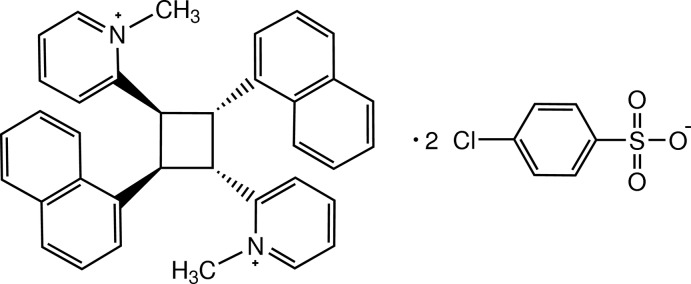


## Experimental   

### 

#### Crystal data  

C_36_H_32_N_2_^2+^·2C_6_H_4_ClO_3_S^−^
*M* *_r_* = 875.86
Triclinic, 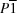

*a* = 7.5488 (3) Å
*b* = 11.1899 (4) Å
*c* = 12.3853 (5) Å
α = 79.904 (2)°
β = 75.964 (2)°
γ = 89.266 (2)°
*V* = 998.76 (7) Å^3^
*Z* = 1
Mo *K*α radiation
μ = 0.32 mm^−1^
*T* = 100 K
0.56 × 0.50 × 0.21 mm

#### Data collection  

Bruker APEXII CCD area-detector diffractometer
Absorption correction: multi-scan (*SADABS*; Bruker, 2005 ▶) *T*_min_ = 0.837, *T*_max_ = 0.936
35475 measured reflections
5817 independent reflections
4977 reflections with *I* > 2σ(*I*)
*R*_int_ = 0.031

#### Refinement  

*R*[*F* ^2^ > 2σ(*F* ^2^)] = 0.040
*wR*(*F* ^2^) = 0.115
*S* = 1.09
5817 reflections
351 parameters
All H-atom parameters refined
Δρ_max_ = 0.50 e Å^−3^
Δρ_min_ = −0.74 e Å^−3^

### 

Data collection: *APEX2* (Bruker, 2005 ▶); cell refinement: *SAINT* (Bruker, 2005 ▶); data reduction: *SAINT*; program(s) used to solve structure: *SHELXTL* (Sheldrick, 2008 ▶); program(s) used to refine structure: *SHELXTL*; molecular graphics: *SHELXTL*; software used to prepare material for publication: *SHELXTL*, *PLATON* (Spek, 2009 ▶), *Mercury* (Macrae *et al.*, 2006 ▶) and *publCIF* (Westrip, 2010 ▶).

## Supplementary Material

Crystal structure: contains datablock(s) global, I. DOI: 10.1107/S160053681400645X/rz5110sup1.cif

Structure factors: contains datablock(s) I. DOI: 10.1107/S160053681400645X/rz5110Isup2.hkl

Supporting information file. DOI: 10.1107/S160053681400645X/rz5110Isup3.cml

CCDC reference: 993267

Additional supporting information:  crystallographic information; 3D view; checkCIF report

## Figures and Tables

**Table 1:** Hydrogen-bond geometry (Å, °) *Cg*4 is the centroid of the C1–C6 ring.

*D*—H⋯*A*	*D*—H	H⋯*A*	*D*⋯*A*	*D*—H⋯*A*
C7—H7⋯O3	0.97 (2)	2.51 (2)	3.3762 (18)	147.9 (18)
C17—H17⋯O2^i^	0.974 (17)	2.506 (18)	3.3001 (18)	138.6 (13)
C20—H20⋯O2	0.97 (2)	2.20 (2)	3.1329 (18)	160.5 (19)
C23—H23⋯O1^ii^	0.94 (2)	2.41 (2)	3.1554 (17)	135.8 (18)
C24—H24*B*⋯O1^ii^	0.95 (2)	2.57 (2)	3.2009 (18)	124.3 (16)
C9—H9⋯*Cg*4^iii^	1.00 (2)	2.98 (2)	3.4790 (16)	112.1 (16)

Symmetry codes: (i) 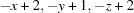; (ii) 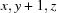; (iii) 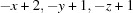.

**Fig. 1.**
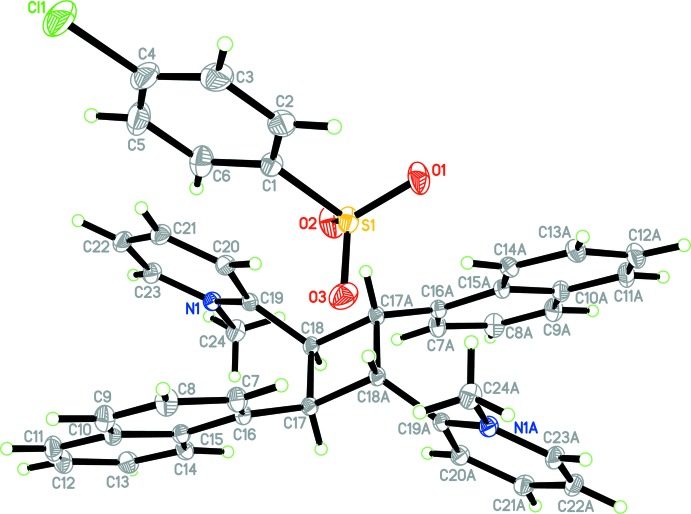
The molecular structure of the title compound, with 50% probability displacement ellipsoids. Symmetry code: (A) 2-x, 1-y, 2-z,

**Fig. 2.**
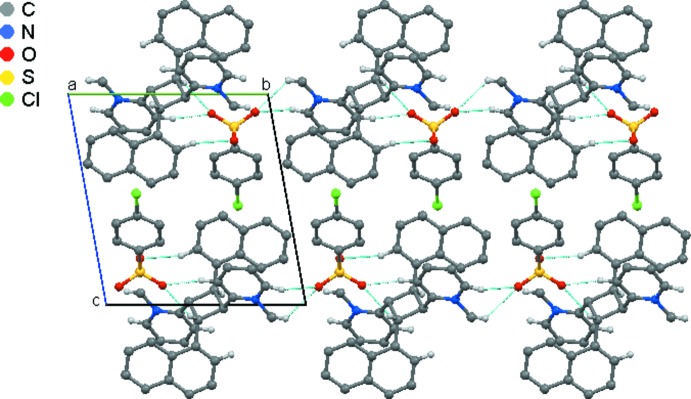
The crystal packing of the title compound viewed down the a axis. H atoms not involved in C—H···O interactions (dashed lines) are omitted for clarity.

**Fig. 3.**
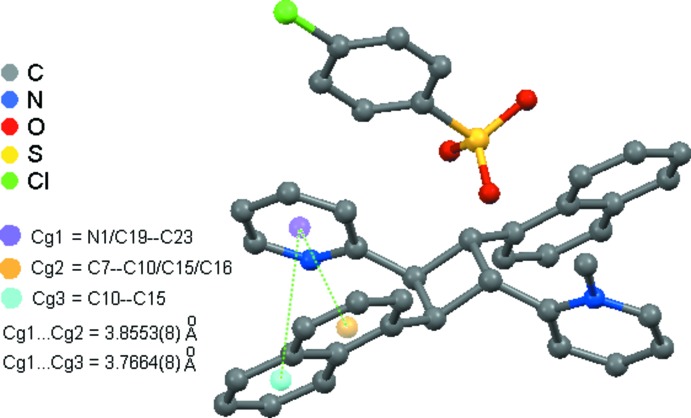
The π···π stacking interactions between the pyridinium and napthalene rings. H atoms are omitted for clarity.

## supplementary crystallographic information

### 1. Comment

Stilbene derivatives have been reported to exhibit non-linear optical (NLO) property (Ruanwas *et al.*, 2010) and antibacterial activity (Chanawanno *et al.*, 2010). It has been known that [2 + 2] photodimerization of stilbenes to yield cyclobutane can occur (Papaefstathiou *et al.*, 2002). In our cases, the [2 + 2] cycloaddition of heterostilbene derivatives was carried out in solution by thermal-induced cycloaddition reaction, and we have previously reported the crystal structures of some of these derivatives (Chantrapromma *et al.*, 2012; Fun, Chanawanno & Chantrapromma, 2009; Fun, Surasit *et al.*, 2009). The title compound (I) was obtained by the cycloaddition of *trans*-heterostilbene to give a *syn* head-to-tail product (Yayli *et al.*, 2004; Zhang *et al.*, 2013). We report herein the synthesis and crystal structure of (I).

The asymmetric unit of (I), C_36_H_32_N_2_^2+^·2(C_6_H_4_ClO_3_S^-^), consists of one half of a cation and one anion. The cation lies on an inversion center and the other half is generated by the symmetry operator 2-*x*, 1-*y*, 2-*z* (Fig. 1). The napthalene (C7–C16) moiety is planar with a *r.m.s.* of 0.0183 (2) Å. The dihedral angle between the pyridinium ring (N1/C19–C23) and the napthalene ring system is 42.86 (6)°. The steroisomer of (I) is *syn* head-to-tail (Yayli *et al.*, 2004). The cyclobutane ring makes dihedral angles of 85.61 (8) and 52.8 (6)° with the pyridinium and naphthalene rings, respectively. The bond lengths are in normal ranges (Allen *et al.*, 1987) and comparable with those found in closely related structures (Chantrapromma *et al.*, 2012; Fun, Surasit *et al.*, 2009; Fun, Chanawanno & Chantrapromma (2009).

The crystal packing of (I) is shown in Fig. 2. The cations and anions are alternatively arranged and linked into chains along the [0 1 0] direction through C—H···O weak interactions (Table 1). Adjacent chains are arranged in a anti-parallel manner into sheets parallel to the (1 0 0) plane. π···π interactions are present with distances of *Cg*_1_···*Cg*_2_ = 3.8553 (8) Å and *Cg*_1_···*Cg*_3_ = 3.7664 (8) Å; *Cg*_1_, *Cg*_2_ and *Cg*_3_ are the centroids of the N1/C19–C23, C7–C10/C15/C16 and C10–C15 rings, respectively (Fig. 3). C—H···π weak interactions are also observed (Table 1).

### 2. Experimental

A solution of (*E*)-1-methyl-2-[2-(1-naphthyl)vinyl)pyridinium iodide (0.25 g, 0.67 mmol) in CH_3_OH (20 ml) was mixed (1:1 molar ratio) with a solution of silver(I) 4-chlorobenzenesulfonate (0.20 g, 0.67 mmol) (Chantrapromma *et al.*, 2007) in CH_3_OH (80 ml) and stirred for 30 min. The precipitate of silver iodide which formed was filtered and the filtrate was evaporated to give a yellow solid product. The yellow solid was repeatedly recrystallized for three times by dissolving the yellow solid in CH_3_OH and the solution was heated at 323 K to get a clear solution. The [2 + 2] cycloaddition of (*E*)-1-methyl-2-[2-(1-naphthyl)vinyl)pyridinium occurred upon heating. Yellow plate-shaped single crystals of the title compound suitable for *X*-ray structure determination were obtained after recrystallization in CH_3_OH by slow evaporation of the solvent at room temperature after a few weeks.

### 3. Refinement

All H atoms were located in difference Fourier map and refined isotropically.

### Crystal data

**Table:**

C_36_H_32_N_2_^2+^·2C_6_H_4_ClO_3_S^−^	*Z* = 1
*M**_r_* = 875.86	*F*(000) = 456
Triclinic, *P*1	*D*_x_ = 1.456 Mg m^−^^3^
Hall symbol: -P 1	Mo *K*α radiation, λ = 0.71073 Å
*a* = 7.5488 (3) Å	Cell parameters from 5817 reflections
*b* = 11.1899 (4) Å	θ = 1.9–30.0°
*c* = 12.3853 (5) Å	µ = 0.32 mm^−^^1^
α = 79.904 (2)°	*T* = 100 K
β = 75.964 (2)°	Plate, yellow
γ = 89.266 (2)°	0.56 × 0.50 × 0.21 mm
*V* = 998.76 (7) Å^3^	

### Data collection

**Table:**

Bruker APEXII CCD area-detector diffractometer	5817 independent reflections
Radiation source: sealed tube	4977 reflections with *I* > 2σ(*I*)
Graphite monochromator	*R*_int_ = 0.031
φ and ω scans	θ_max_ = 30.0°, θ_min_ = 1.9°
Absorption correction: multi-scan (*SADABS*; Bruker, 2005)	*h* = −10→10
*T*_min_ = 0.837, *T*_max_ = 0.936	*k* = −15→15
35475 measured reflections	*l* = −17→17

### Refinement

**Table:**

Refinement on *F*^2^	Primary atom site location: structure-invariant direct methods
Least-squares matrix: full	Secondary atom site location: difference Fourier map
*R*[*F*^2^ > 2σ(*F*^2^)] = 0.040	Hydrogen site location: inferred from neighbouring sites
*wR*(*F*^2^) = 0.115	All H-atom parameters refined
*S* = 1.09	*w* = 1/[σ^2^(*F*_o_^2^) + (0.052*P*)^2^ + 0.7427*P*] where *P* = (*F*_o_^2^ + 2*F*_c_^2^)/3
5817 reflections	(Δ/σ)_max_ = 0.001
351 parameters	Δρ_max_ = 0.50 e Å^−^^3^
0 restraints	Δρ_min_ = −0.74 e Å^−^^3^

### Special details

**Table:**

Experimental. The crystal was placed in the cold stream of an Oxford Cryosystems Cobra open-flow nitrogen cryostat (Cosier & Glazer, 1986) operating at 100.0 (1) K.
Geometry. All e.s.d.'s (except the e.s.d. in the dihedral angle between two l.s. planes) are estimated using the full covariance matrix. The cell e.s.d.'s are taken into account individually in the estimation of e.s.d.'s in distances, angles and torsion angles; correlations between e.s.d.'s in cell parameters are only used when they are defined by crystal symmetry. An approximate (isotropic) treatment of cell e.s.d.'s is used for estimating e.s.d.'s involving l.s. planes.
Refinement. Refinement of *F*^2^ against ALL reflections. The weighted *R*-factor *wR* and goodness of fit *S* are based on *F*^2^, conventional *R*-factors *R* are based on *F*, with *F* set to zero for negative *F*^2^. The threshold expression of *F*^2^ > σ(*F*^2^) is used only for calculating *R*-factors(gt) *etc*. and is not relevant to the choice of reflections for refinement. *R*-factors based on *F*^2^ are statistically about twice as large as those based on *F*, and *R*- factors based on ALL data will be even larger.

### Fractional atomic coordinates and isotropic or equivalent isotropic displacement parameters (Å^2^)

**Table:**

	*x*	*y*	*z*	*U*_iso_*/*U*_eq_	
Cl1	0.34039 (7)	0.25081 (7)	0.47197 (4)	0.05223 (17)	
S1	0.78639 (5)	0.19952 (3)	0.84360 (3)	0.01743 (9)	
O1	0.73608 (18)	0.08040 (9)	0.91361 (10)	0.0249 (2)	
O2	0.73144 (17)	0.29857 (10)	0.90582 (9)	0.0247 (2)	
O3	0.97566 (16)	0.21127 (10)	0.77961 (10)	0.0245 (2)	
N1	0.71757 (16)	0.75327 (10)	0.97380 (10)	0.0141 (2)	
C1	0.65583 (19)	0.21394 (13)	0.74055 (12)	0.0169 (2)	
C2	0.6222 (2)	0.11222 (14)	0.69692 (13)	0.0216 (3)	
H2	0.669 (3)	0.036 (2)	0.7248 (18)	0.030 (5)*	
C3	0.5228 (2)	0.12351 (18)	0.61444 (14)	0.0295 (4)	
H3	0.499 (4)	0.050 (2)	0.587 (2)	0.048 (7)*	
C4	0.4605 (2)	0.23620 (19)	0.57678 (13)	0.0310 (4)	
C5	0.4931 (2)	0.33809 (18)	0.61922 (14)	0.0306 (4)	
H5	0.445 (4)	0.416 (2)	0.594 (2)	0.044 (7)*	
C6	0.5919 (2)	0.32641 (15)	0.70183 (13)	0.0243 (3)	
H6	0.614 (3)	0.399 (2)	0.733 (2)	0.043 (7)*	
C7	1.1080 (2)	0.50647 (12)	0.73600 (12)	0.0171 (3)	
H7	1.107 (3)	0.420 (2)	0.7661 (18)	0.031 (5)*	
C8	1.0998 (2)	0.54409 (13)	0.62185 (12)	0.0201 (3)	
H8	1.094 (3)	0.4832 (19)	0.5774 (17)	0.023 (5)*	
C9	1.0930 (2)	0.66488 (14)	0.57787 (12)	0.0198 (3)	
H9	1.083 (3)	0.692 (2)	0.498 (2)	0.037 (6)*	
C10	1.09915 (19)	0.75385 (12)	0.64568 (11)	0.0166 (2)	
C11	1.0880 (2)	0.87977 (13)	0.60332 (12)	0.0201 (3)	
H11	1.079 (3)	0.9014 (19)	0.5266 (17)	0.025 (5)*	
C12	1.0923 (2)	0.96528 (13)	0.66949 (13)	0.0214 (3)	
H12	1.081 (3)	1.0496 (19)	0.6386 (17)	0.025 (5)*	
C13	1.1119 (2)	0.92916 (12)	0.78099 (13)	0.0187 (3)	
H13	1.113 (3)	0.9900 (19)	0.8269 (17)	0.024 (5)*	
C14	1.12389 (19)	0.80819 (12)	0.82451 (12)	0.0156 (2)	
H14	1.139 (3)	0.7860 (17)	0.9006 (16)	0.016 (4)*	
C15	1.11430 (18)	0.71681 (12)	0.75936 (11)	0.0139 (2)	
C16	1.11371 (18)	0.58955 (12)	0.80500 (11)	0.0136 (2)	
C17	1.10424 (18)	0.55360 (11)	0.92961 (11)	0.0127 (2)	
H17	1.208 (2)	0.5887 (16)	0.9483 (15)	0.013 (4)*	
C18	0.92111 (18)	0.58354 (11)	1.01586 (11)	0.0128 (2)	
H18	0.951 (3)	0.6318 (18)	1.0651 (17)	0.022 (5)*	
C19	0.77448 (17)	0.63876 (11)	0.96192 (11)	0.0127 (2)	
C20	0.70618 (18)	0.58062 (12)	0.88915 (12)	0.0154 (2)	
H20	0.743 (3)	0.4980 (18)	0.8833 (17)	0.023 (5)*	
C21	0.59784 (19)	0.64167 (13)	0.82278 (12)	0.0175 (3)	
H21	0.552 (3)	0.6025 (19)	0.7721 (18)	0.027 (5)*	
C22	0.5555 (2)	0.76146 (13)	0.83035 (13)	0.0187 (3)	
H22	0.478 (3)	0.806 (2)	0.7852 (18)	0.028 (5)*	
C23	0.61171 (19)	0.81340 (12)	0.90961 (12)	0.0174 (3)	
H23	0.578 (3)	0.8907 (19)	0.9267 (17)	0.025 (5)*	
C24	0.7691 (2)	0.81744 (13)	1.05723 (12)	0.0178 (3)	
H24A	0.750 (3)	0.7626 (19)	1.1291 (17)	0.023 (5)*	
H24B	0.694 (3)	0.886 (2)	1.0634 (18)	0.031 (6)*	
H24C	0.895 (3)	0.8424 (19)	1.0323 (18)	0.027 (5)*	

### Atomic displacement parameters (Å^2^)

**Table:**

	*U*^11^	*U*^22^	*U*^33^	*U*^12^	*U*^13^	*U*^23^
Cl1	0.0367 (3)	0.0909 (5)	0.0291 (2)	−0.0113 (3)	−0.02130 (19)	0.0088 (2)
S1	0.02519 (18)	0.00977 (15)	0.02051 (17)	−0.00007 (12)	−0.01122 (13)	−0.00329 (11)
O1	0.0397 (6)	0.0112 (4)	0.0270 (5)	−0.0022 (4)	−0.0167 (5)	0.0003 (4)
O2	0.0404 (7)	0.0139 (5)	0.0229 (5)	−0.0003 (4)	−0.0111 (5)	−0.0072 (4)
O3	0.0231 (5)	0.0204 (5)	0.0343 (6)	0.0009 (4)	−0.0135 (5)	−0.0069 (4)
N1	0.0151 (5)	0.0097 (5)	0.0181 (5)	0.0009 (4)	−0.0051 (4)	−0.0023 (4)
C1	0.0177 (6)	0.0167 (6)	0.0171 (6)	0.0012 (5)	−0.0057 (5)	−0.0030 (5)
C2	0.0245 (7)	0.0209 (7)	0.0214 (7)	−0.0032 (5)	−0.0083 (5)	−0.0049 (5)
C3	0.0287 (8)	0.0401 (10)	0.0219 (7)	−0.0094 (7)	−0.0096 (6)	−0.0057 (7)
C4	0.0197 (7)	0.0538 (11)	0.0184 (7)	−0.0029 (7)	−0.0081 (6)	0.0016 (7)
C5	0.0262 (8)	0.0377 (9)	0.0245 (7)	0.0105 (7)	−0.0072 (6)	0.0040 (7)
C6	0.0284 (8)	0.0219 (7)	0.0224 (7)	0.0073 (6)	−0.0073 (6)	−0.0021 (6)
C7	0.0210 (6)	0.0130 (6)	0.0174 (6)	0.0022 (5)	−0.0050 (5)	−0.0024 (5)
C8	0.0263 (7)	0.0181 (6)	0.0176 (6)	0.0027 (5)	−0.0069 (5)	−0.0057 (5)
C9	0.0236 (7)	0.0203 (7)	0.0154 (6)	0.0030 (5)	−0.0060 (5)	−0.0019 (5)
C10	0.0176 (6)	0.0149 (6)	0.0162 (6)	0.0021 (5)	−0.0043 (5)	0.0000 (5)
C11	0.0238 (7)	0.0164 (6)	0.0185 (6)	0.0030 (5)	−0.0062 (5)	0.0023 (5)
C12	0.0241 (7)	0.0131 (6)	0.0246 (7)	0.0015 (5)	−0.0050 (5)	0.0018 (5)
C13	0.0204 (6)	0.0118 (6)	0.0234 (7)	−0.0002 (5)	−0.0055 (5)	−0.0016 (5)
C14	0.0166 (6)	0.0119 (6)	0.0177 (6)	−0.0008 (4)	−0.0044 (5)	−0.0012 (5)
C15	0.0141 (5)	0.0111 (5)	0.0161 (6)	0.0004 (4)	−0.0042 (4)	−0.0006 (4)
C16	0.0141 (5)	0.0114 (5)	0.0150 (5)	0.0008 (4)	−0.0041 (4)	−0.0007 (4)
C17	0.0144 (5)	0.0086 (5)	0.0153 (5)	0.0006 (4)	−0.0048 (4)	−0.0013 (4)
C18	0.0142 (5)	0.0091 (5)	0.0155 (5)	0.0009 (4)	−0.0053 (4)	−0.0009 (4)
C19	0.0130 (5)	0.0087 (5)	0.0161 (6)	0.0006 (4)	−0.0038 (4)	−0.0010 (4)
C20	0.0163 (6)	0.0109 (5)	0.0197 (6)	0.0001 (4)	−0.0059 (5)	−0.0027 (5)
C21	0.0176 (6)	0.0155 (6)	0.0215 (6)	−0.0010 (5)	−0.0087 (5)	−0.0029 (5)
C22	0.0174 (6)	0.0153 (6)	0.0246 (7)	0.0021 (5)	−0.0097 (5)	−0.0007 (5)
C23	0.0172 (6)	0.0112 (5)	0.0244 (7)	0.0035 (5)	−0.0074 (5)	−0.0017 (5)
C24	0.0225 (7)	0.0129 (6)	0.0204 (6)	0.0033 (5)	−0.0077 (5)	−0.0063 (5)

### Geometric parameters (Å, º)

**Table:**

Cl1—C4	1.7397 (16)	C11—C12	1.370 (2)
S1—O3	1.4511 (12)	C11—H11	0.96 (2)
S1—O1	1.4564 (11)	C12—C13	1.412 (2)
S1—O2	1.4584 (11)	C12—H12	0.96 (2)
S1—C1	1.7776 (14)	C13—C14	1.3769 (18)
N1—C23	1.3529 (17)	C13—H13	0.96 (2)
N1—C19	1.3673 (16)	C14—C15	1.4217 (18)
N1—C24	1.4823 (17)	C14—H14	0.965 (19)
C1—C6	1.390 (2)	C15—C16	1.4368 (17)
C1—C2	1.394 (2)	C16—C17	1.5094 (18)
C2—C3	1.395 (2)	C17—C18^i^	1.5585 (17)
C2—H2	0.96 (2)	C17—C18	1.6005 (18)
C3—C4	1.383 (3)	C17—H17	0.974 (19)
C3—H3	0.97 (3)	C18—C19	1.5000 (18)
C4—C5	1.384 (3)	C18—C17^i^	1.5585 (17)
C5—C6	1.394 (2)	C18—H18	0.95 (2)
C5—H5	0.98 (3)	C19—C20	1.3932 (18)
C6—H6	0.99 (3)	C20—C21	1.3879 (19)
C7—C16	1.3761 (18)	C20—H20	0.97 (2)
C7—C8	1.4189 (19)	C21—C22	1.388 (2)
C7—H7	0.98 (2)	C21—H21	0.95 (2)
C8—C9	1.371 (2)	C22—C23	1.375 (2)
C8—H8	0.96 (2)	C22—H22	0.98 (2)
C9—C10	1.418 (2)	C23—H23	0.94 (2)
C9—H9	1.00 (2)	C24—H24A	0.97 (2)
C10—C11	1.4237 (19)	C24—H24B	0.95 (2)
C10—C15	1.4282 (18)	C24—H24C	0.96 (2)
			
O3—S1—O1	113.75 (7)	C14—C13—C12	120.46 (13)
O3—S1—O2	113.02 (7)	C14—C13—H13	120.5 (12)
O1—S1—O2	112.70 (7)	C12—C13—H13	119.0 (12)
O3—S1—C1	105.26 (7)	C13—C14—C15	121.07 (13)
O1—S1—C1	105.65 (7)	C13—C14—H14	118.8 (11)
O2—S1—C1	105.50 (7)	C15—C14—H14	120.1 (11)
C23—N1—C19	121.43 (12)	C14—C15—C10	118.30 (12)
C23—N1—C24	116.89 (11)	C14—C15—C16	122.34 (12)
C19—N1—C24	121.68 (11)	C10—C15—C16	119.33 (12)
C6—C1—C2	120.35 (14)	C7—C16—C15	118.98 (12)
C6—C1—S1	120.19 (11)	C7—C16—C17	123.07 (12)
C2—C1—S1	119.44 (11)	C15—C16—C17	117.77 (11)
C1—C2—C3	119.68 (15)	C16—C17—C18^i^	118.70 (11)
C1—C2—H2	118.5 (13)	C16—C17—C18	117.06 (11)
C3—C2—H2	121.8 (13)	C18^i^—C17—C18	90.59 (9)
C4—C3—C2	119.18 (16)	C16—C17—H17	110.6 (11)
C4—C3—H3	122.9 (16)	C18^i^—C17—H17	109.6 (11)
C2—C3—H3	117.9 (16)	C18—C17—H17	108.5 (10)
C3—C4—C5	121.80 (15)	C19—C18—C17^i^	117.10 (11)
C3—C4—Cl1	119.17 (15)	C19—C18—C17	115.18 (11)
C5—C4—Cl1	119.03 (15)	C17^i^—C18—C17	89.41 (9)
C4—C5—C6	118.86 (16)	C19—C18—H18	111.7 (12)
C4—C5—H5	120.9 (15)	C17^i^—C18—H18	112.1 (12)
C6—C5—H5	120.3 (15)	C17—C18—H18	109.4 (12)
C1—C6—C5	120.13 (16)	N1—C19—C20	117.97 (12)
C1—C6—H6	120.6 (15)	N1—C19—C18	120.58 (11)
C5—C6—H6	119.2 (15)	C20—C19—C18	121.12 (11)
C16—C7—C8	121.32 (13)	C21—C20—C19	120.72 (12)
C16—C7—H7	119.8 (13)	C21—C20—H20	121.6 (12)
C8—C7—H7	118.9 (13)	C19—C20—H20	117.6 (12)
C9—C8—C7	120.64 (13)	C20—C21—C22	119.42 (13)
C9—C8—H8	120.8 (12)	C20—C21—H21	120.9 (13)
C7—C8—H8	118.5 (12)	C22—C21—H21	119.6 (13)
C8—C9—C10	120.03 (13)	C23—C22—C21	118.63 (13)
C8—C9—H9	120.9 (14)	C23—C22—H22	120.2 (13)
C10—C9—H9	119.0 (14)	C21—C22—H22	121.0 (13)
C9—C10—C11	121.36 (13)	N1—C23—C22	121.37 (13)
C9—C10—C15	119.59 (12)	N1—C23—H23	114.0 (13)
C11—C10—C15	119.04 (13)	C22—C23—H23	124.6 (13)
C12—C11—C10	121.14 (13)	N1—C24—H24A	109.0 (12)
C12—C11—H11	122.0 (12)	N1—C24—H24B	107.3 (14)
C10—C11—H11	116.8 (12)	H24A—C24—H24B	111.1 (18)
C11—C12—C13	119.94 (13)	N1—C24—H24C	109.9 (13)
C11—C12—H12	118.9 (12)	H24A—C24—H24C	108.7 (18)
C13—C12—H12	121.2 (12)	H24B—C24—H24C	110.7 (19)
			
O3—S1—C1—C6	−93.88 (13)	C11—C10—C15—C16	176.00 (12)
O1—S1—C1—C6	145.45 (13)	C8—C7—C16—C15	−1.0 (2)
O2—S1—C1—C6	25.87 (14)	C8—C7—C16—C17	173.93 (13)
O3—S1—C1—C2	84.49 (13)	C14—C15—C16—C7	−178.65 (13)
O1—S1—C1—C2	−36.18 (14)	C10—C15—C16—C7	3.31 (19)
O2—S1—C1—C2	−155.76 (12)	C14—C15—C16—C17	6.13 (19)
C6—C1—C2—C3	−0.4 (2)	C10—C15—C16—C17	−171.91 (12)
S1—C1—C2—C3	−178.75 (12)	C7—C16—C17—C18^i^	−2.56 (19)
C1—C2—C3—C4	0.5 (2)	C15—C16—C17—C18^i^	172.45 (11)
C2—C3—C4—C5	−0.4 (3)	C7—C16—C17—C18	−109.58 (14)
C2—C3—C4—Cl1	178.78 (13)	C15—C16—C17—C18	65.43 (15)
C3—C4—C5—C6	0.2 (3)	C16—C17—C18—C19	3.08 (16)
Cl1—C4—C5—C6	−178.95 (13)	C18^i^—C17—C18—C19	−119.90 (13)
C2—C1—C6—C5	0.2 (2)	C16—C17—C18—C17^i^	122.98 (13)
S1—C1—C6—C5	178.57 (13)	C18^i^—C17—C18—C17^i^	0.0
C4—C5—C6—C1	−0.1 (3)	C23—N1—C19—C20	−6.00 (19)
C16—C7—C8—C9	−1.5 (2)	C24—N1—C19—C20	174.15 (12)
C7—C8—C9—C10	1.7 (2)	C23—N1—C19—C18	167.49 (12)
C8—C9—C10—C11	−178.48 (14)	C24—N1—C19—C18	−12.36 (19)
C8—C9—C10—C15	0.6 (2)	C17^i^—C18—C19—N1	139.98 (12)
C9—C10—C11—C12	179.41 (14)	C17—C18—C19—N1	−116.85 (13)
C15—C10—C11—C12	0.3 (2)	C17^i^—C18—C19—C20	−46.74 (17)
C10—C11—C12—C13	1.3 (2)	C17—C18—C19—C20	56.43 (16)
C11—C12—C13—C14	−1.0 (2)	N1—C19—C20—C21	6.1 (2)
C12—C13—C14—C15	−0.9 (2)	C18—C19—C20—C21	−167.36 (13)
C13—C14—C15—C10	2.5 (2)	C19—C20—C21—C22	−0.8 (2)
C13—C14—C15—C16	−175.60 (13)	C20—C21—C22—C23	−4.6 (2)
C9—C10—C15—C14	178.74 (13)	C19—N1—C23—C22	0.6 (2)
C11—C10—C15—C14	−2.1 (2)	C24—N1—C23—C22	−179.56 (13)
C9—C10—C15—C16	−3.1 (2)	C21—C22—C23—N1	4.8 (2)

Symmetry code: (i) −*x*+2, −*y*+1, −*z*+2.

### Hydrogen-bond geometry (Å, º)

*Cg*4 is the centroid of the C1–C6 ring.

**Table:**

*D*—H···*A*	*D*—H	H···*A*	*D*···*A*	*D*—H···*A*
C7—H7···O3	0.97 (2)	2.51 (2)	3.3762 (18)	147.9 (18)
C17—H17···O2^i^	0.974 (17)	2.506 (18)	3.3001 (18)	138.6 (13)
C20—H20···O2	0.97 (2)	2.20 (2)	3.1329 (18)	160.5 (19)
C23—H23···O1^ii^	0.94 (2)	2.41 (2)	3.1554 (17)	135.8 (18)
C24—H24*B*···O1^ii^	0.95 (2)	2.57 (2)	3.2009 (18)	124.3 (16)
C9—H9···*Cg*4^iii^	1.00 (2)	2.98 (2)	3.4790 (16)	112.1 (16)

Symmetry codes: (i) −*x*+2, −*y*+1, −*z*+2; (ii) *x*, *y*+1, *z*; (iii) −*x*+2, −*y*+1, −*z*+1.
